# Perfluoroalkyl Substances, Sex Hormones, and Insulin-like Growth Factor-1 at 6–9 Years of Age: A Cross-Sectional Analysis within the C8 Health Project

**DOI:** 10.1289/ehp.1509869

**Published:** 2016-01-22

**Authors:** Maria-Jose Lopez-Espinosa, Debapriya Mondal, Ben G. Armstrong, Brenda Eskenazi, Tony Fletcher

**Affiliations:** 1 Department of Social and Environmental Health Research, London School of Hygiene & Tropical Medicine, London, United Kingdom; 2 Epidemiology and Environmental Health Joint Research Unit, FISABIO (Foundation for the Promotion of Health and Biomedical Research in the Valencian Region)–Universitat Jaume I–Universitat de València, Valencia, Spain; 3 Spanish Consortium for Research on Epidemiology and Public Health (CIBERESP), Madrid, Spain; 4 School of Environment and Life Sciences, University of Salford, The Crescent, Salford, United Kingdom; 5 Center for Environmental Research and Children’s Health (CERCH), School of Public Health, University of California, Berkeley, Berkeley, California, USA

## Abstract

**Background:**

Exposure to some perfluoroalkyl substances (PFAS), such as perfluorohexane sulfonate (PFHxS), perfluorooctanoate (PFOA), perfluorooctane sulfonate (PFOS), and perfluorononanoic acid (PFNA), may alter levels of sex hormones and insulin-like growth factor-1 (IGF-1) in animals. Human studies on this topic are scarce, and none have been conducted in young children.

**Objectives:**

We investigated the relationship between levels of PFAS and estradiol, total testosterone, and IGF-1 in 2,292 children (6–9 years of age) from the C8 Health Project who lived near a chemical plant in the Mid-Ohio Valley (USA) with local contamination from PFOA.

**Methods:**

Serum samples were collected in 2005–2006 and analyzed for PFAS, sex hormones, and IGF-1. Results from regression models were expressed as the adjusted percentage difference (95% CI) per sex-specific interquartile range (IQR) increment of each PFAS serum concentration. Analyses by PFAS quartiles were also conducted.

**Results:**

Median concentrations of PFHxS, PFOA, PFOS, and PFNA were 8, 35, 22, and 1.7 ng/mL in boys and 7, 30, 21, and 1.7 ng/mL in girls. In boys, PFOA concentrations were significantly associated with testosterone levels (–4.9%; 95% CI: –8.7, –0.8%); PFOS with estradiol (–4.0%; 95% CI: –7.7, –0.1%), testosterone (–5.8%; 95% CI: –9.4, –2.0%), and IGF-1 (–5.9%; 95% CI: –8.3, –3.3%); and PFNA with IGF-1 (–3.5%; 95% CI: –6.0, –1.0%). In girls, significant associations were found between PFOS and testosterone (–6.6%; 95% CI: –10.1, –2.8%) and IGF-1 (–5.6%; –8.2, –2.9%); and PFNA and IGF-1 (–3.8%; 95% CI: –6.4, –1.2%). In both sexes, the magnitudes of the associations decreased monotonically across quartiles for both testosterone and IGF-1 in relation to PFOS, and for IGF-1 and PFNA in girls.

**Conclusions:**

To our knowledge, this is the first study suggesting that PFAS are associated with lower levels of IGF-1 and sex hormones in young children.

**Citation:**

Lopez-Espinosa MJ, Mondal D, Armstrong BG, Eskenazi B, Fletcher T. 2016. Perfluoroalkyl substances, sex hormones, and insulin-like growth factor-1 at 6–9 years of age: a cross-sectional analysis within the C8 Health Project. Environ Health Perspect 124:1269–1275; http://dx.doi.org/10.1289/ehp.1509869

## Introduction

Perfluorohexane sulfonate (PFHxS), perfluorooctanoate (PFOA), perfluorooctane sulfonate (PFOS), and perfluorononanoic acid (PFNA) are members of the class of human-made perfluoroalkyl substances (PFAS) that are widely used in household and commercial products (Renner 2001; WHO 2013). Studies around the world have shown measurable levels of these four PFAS in the serum of the studied populations (Fromme et al. 2009; Halldorsson et al. 2012; Kato et al. 2011; Mondal et al. 2012) and, given their long half-life in humans (Bartell et al. 2010), exposure to these compounds will persist for many years.

Even though humans are exposed to PFAS and they may disrupt the endocrine system (Jensen and Leffers 2008), little is known about their possible health effects. In some animal studies exposure to these contaminants has been linked with alterations in levels of sex hormones (Lau et al. 2007; Wan et al. 2011). However, few epidemiological data have been published on this topic despite the key roles played by sex hormones in developmental and reproductive functions during childhood and puberty (Norgil Damgaard et al. 2002; Schoeters et al. 2008). In a cross-sectional study of boys 8–18 years of age in the Mid-Ohio Valley, we reported a 6-month delay in the average age at which the boys entered puberty (using testosterone levels > 50 ng/dL as an indicator of having started puberty), comparing the highest and the lowest PFOS quartiles (Lopez-Espinosa et al. 2011). A study on Taiwanese boys 12–17 years of age (Tsai et al. 2015) reported an inverse association between PFOS and follicle-stimulating hormone (FSH), but found no significant associations between PFAS and estrogen, testosterone, luteinizing hormone (LH), or sex hormone–binding globulin (SHBG). Information on adult male populations is also scarce and inconclusive. PFOS and PFNA were associated inversely with testosterone and estradiol (Joensen et al. 2013) and PFOA positively with LH and FSH (Vested et al. 2013) in young Danish men with a mean age of around 20 years. In male American adults 30–66 years of age, positive associations between both PFOA and PFOS and LH were also reported (Raymer et al. 2012). Other studies, however, did not find such statistically significant associations (Olsen et al. 1998; Specht et al. 2012).

The relationship between PFAS and female reproductive hormones is not clear either. In girls 15 years of age from the ALSPAC (Avon Longitudinal Study of Parents and Children) cohort (UK), positive associations were found between prenatal PFHxS, PFOA, and PFOS exposure and testosterone (Maisonet et al. 2015). In contrast, inverse associations were found between PFOS and testosterone in Taiwanese girls 12–17 years of age (Tsai et al. 2015), but no significant associations were reported between prenatal PFOA or PFOS exposure and estradiol or testosterone in 20-year-old Danish women (Kristensen et al. 2013). In addition, an inverse association between PFOS and estradiol levels was reported among perimenopausal and menopausal women in the Mid-Ohio Valley (Knox et al. 2011). However, an explanation for the pattern found by Knox et al. (2011) may be reverse causality through menopause, leading to lower excretion rates (via menstrual blood loss) and thus higher PFAS concentrations in serum (Konkel 2014; Taylor et al. 2014). Other hormones and proteins involved in sexual maturation have also been studied in the previous studies. SHBG levels were inversely associated with PFOA in girls 12–17 years of age from the Taiwanese cohort (Tsai et al. 2015), but such significant association was not observed in 15-year-old girls from the UK cohort (Maisonet et al. 2015). Also, no statistically significant associations were found between PFAS and LH or FSH in adolescent girls (Kristensen et al. 2013; Tsai et al. 2015).

In addition to sex hormones, insulin-like growth factor-1 (IGF-1) also plays important roles in growth and sexual maturation. Dynamic interactions taking place among growth hormone (GH), IGF-1, and sex hormones increase muscle mass, affect the mineralization of the skeleton, and control growth and metabolic changes during the prepubertal and pubertal periods (Garnett et al. 2004; Le Roith and Butler 1999; Mauras 2001). IGF-1 stimulates testosterone synthesis and maintains normal fertility in males (Wan et al. 2011). It is also involved in the development and maturation of the pubertal mammary gland in females (Kleinberg and Ruan 2008), and has been shown to predict the age of menarche (Thankamony et al. 2012). In addition, IGF-1 levels in childhood and adolescence are predictive of IGF-1 levels in adulthood (Sandhu et al. 2006), and lower levels of IGF-1 in adults have been associated with diabetes, cardiovascular diseases, and an increased risk of mortality (Burgers et al. 2011; Ceda et al. 2005; Vaessen et al. 2001). Well-known factors influencing IGF-1 levels are diet and lifestyle (Baibas et al. 2003; Berg and Bang 2004; Savage 2013), but exposure to endocrine-disrupting chemicals may also alter the normal synthesis and/or secretion of IGF-1 (Zumbado et al. 2010). However, to our knowledge, no data on the association between IGF-1 and PFAS in humans have been published to date.

In this study we aimed to assess the association between serum concentrations of four PFAS (PFHxS, PFOA, PFOS, and PFNA) and total testosterone, estradiol, and IGF-1 in 6- to 9-year-old children from the Mid-Ohio Valley, USA.

## Material and Methods

### Study Participants

Data on 69,030 people were collected in the “C8 Health Project” between August 2005 and July 2006. The details of the study, participants, enrollment criteria, and consent procedures are described elsewhere (Frisbee et al. 2009). PFOA had been used in the manufacture of fluoropolymers at a chemical plant in Parkersburg, West Virginia, since 1951 and led to contamination of local drinking water with PFOA. Individuals were eligible to participate in the C8 Health Project if they had consumed water for at least 1 year between 1950 and 2004 from the six contaminated water districts or private wells in the proximity of the chemical plant.

For this study, we focused on the subset of children 6–9 years of age at enrollment. We set a minimum of 6 years because the proportion of children with hormone levels below the detection limit (LOD) rose sharply below age 6 years, and a maximum of 9 years was established to largely avoid including children who had started their pubertal development. Four boys had entered puberty using the criterion of testosterone levels > 50 ng/dL as a marker (Lopez-Espinosa et al. 2011), and 13 girls reported menarche by the time of the survey. These 17 children (0.7%) were excluded. Of the remaining children, 96% had measures of PFAS, hormones, and/or IGF-1, resulting in a final sample size of 2,292. Parents or guardians provided written informed consent on behalf of the children participating in the present study. The London School of Hygiene and Tropical Medicine Ethics Committee approved this study.

### Determination of PFAS

Laboratory analyses of serum PFAS were conducted by a commercial laboratory (Exygen Research). Samples collected at survey were analyzed for 10 PFAS including PFHxS, PFOA, PFOS, and PFNA. The analytical methods and quality control procedures employed by the laboratory have been described elsewhere (Flaherty et al. 2005; Frisbee et al. 2009). Briefly, the technique used solid-phase extraction followed by reverse-phase high-performance liquid chromatography/mass spectrometry. Over a range of 0.5–40 ng/mL, the coefficient of variation (CV) for PFOA was generally < 10% for multiple samples measured in different batches. CVs for PFHxS, PFOS, and PFNA were similar to those for PFOA. The LOD was 0.5 ng/mL for all of them. A quality assurance program was carried out by analysis of duplicate samples at AXYS Analytical Service Ltd. Details on the interlaboratory reliability have been extensively reported by Frisbee et al. (2009).

### Determination of Sex Hormones and IGF-1

Clinical laboratory tests were performed at an accredited clinical diagnostic laboratory (LabCorp). Estradiol and total testosterone levels were measured in serum samples using an electrochemiluminescence immunoassay (Roche Diagnostics) with LODs of 7 pg/mL and 10 ng/dL, respectively. Free testosterone was measured by a radioimmunoassay in serum (Coat-a-Count; Siemens Healthcare Diagnostics) with an LOD of 0.2 pg/mL. IGF-1 was measured in serum by an immunochemiluminometric assay (Siemens Healthcare Diagnostics) with an LOD of 25 ng/mL. Volumes used for analyses were: 35, 50, 50, and 20 μL for estradiol, total testosterone, free testosterone, and IGF-1, respectively. A total of 33.4% of the blood samples were drawn before 1200 hours, 43.6% between 1200 and 1600 hours, and 23.0% between 1700 and 1900 hours.

### Covariates

Covariates selected *a priori* for the analyses included sex, race/ethnicity (non-Hispanic white and others), age (years), height (inches), weight (pounds), body mass index (BMI; kilograms per meter squared) transformed into a *z*-score based on the 2000 U.S. Centers for Disease Control and Prevention growth charts of height/weight/BMI-for-age (CDC 2010), household annual family income (classified in the original questionnaire as ≤ $20,000; $20,000–70,000; > $70,000; and not known), and the month and hour of sample collection.

### Statistical Analyses

We present descriptive analyses for the three sex hormones and IGF-1, but free testosterone was excluded from the multivariate analyses due to the low proportion of samples above the LOD. Outcomes showed a non-normal distribution and were natural log transformed before inclusion in the models.

PFAS observations below the LOD (< 0.5% for all the PFAS) were replaced by the LOD divided by 2 for descriptive and statistical analyses. PFAS did not show a normal distribution either and were natural log transformed as well as categorized in quartiles for the statistical analyses. We used simple Pearson’s correlations to describe pairwise relationships between ln(PFAS).

We used interval regression for censored data to assess the relation between outcomes and ln(PFAS) or PFAS in quartiles. These models were constructed to provide maximum likelihood estimations of the coefficients in the presence of LODs in the dependent variable (Lubin et al. 2004). All models were adjusted for child’s age and sampling month (because of a trend in serum PFAS during the collection year as well as seasonal variations in levels of hormones and IGF-1). Total testosterone models were additionally adjusted for time of day of blood sampling due to diurnal variations in their levels. Race did not meet our operational definition of confounder because there was < 10% change in the PFAS coefficients when including or excluding it from the final regression models. Analyses were sex-stratified, but we also investigated differences by sex including the interaction of this variable with the contaminants in the joint models (i.e., models including both girls and boys).

For PFAS as continuous variables, we expressed results as percentage difference [with 95% confidence interval (CI)] in the outcomes associated with a sex-specific interquartile range (IQR) increment in PFAS concentrations, and the percentage was calculated as the complement of the exponentiated regression coefficient [100×(exp(β × IQR) – 1)]. For PFAS in quartiles, we expressed results as the percentage difference (95% CI) in outcome across quartiles of exposure (with the lowest as the reference). Tests for trend were conducted across quartiles using a variable with the median concentration of PFAS in each quartile added to models as a numerical regressor. Finally, we also fitted multi-pollutant models including all four ln(PFAS) in the same model.

We used the multivariate imputation procedure by chained equations (Horton and Kleinman 2007; van Buuren and Groothuis-Oudshoorn 2011) to impute missing values according to available information. Variables included in the procedure were eligible for inclusion based on prediction ability (correlation ≥ 0.05), their relation to the nonresponse (correlation ≥ 0.05), and the existence of at least 20% with known values within the subgroup of incomplete cases (van Buuren and Groothuis-Oudshoorn 2011), including the outcome and exposure variables, covariates, and other predictors not included in the main analyses, without interactions (see Table S1). We imputed 50 data sets (20 cycles for each) and assessed convergence by plotting parameters (mean and SD in each imputed data set) against iteration number. In addition, we checked imputations of missing values graphically, and compared them with observed data.

We performed sensitivity analyses with these imputed data. First, we considered the imputed variable household income as a possible confounder in our analyses although it did not meet our operational definition of confounder. Missing values for this variable (*n* = 344, 15%) were imputed according to available data for maternal education, current employment, and mother’s and child’s race (see Table S1). We also reran analyses after excluding individuals with missing household income data, and found no noteworthy changes in the results (data not shown). Second, the main analyses were not adjusted for height or BMI because these variables may be affected by outcomes. However, people who are larger at a given age might have higher IGF-1 levels, and there is some concern that growth (and increased body volume) resulting from higher IGF-1 levels might cause PFAS concentrations to decrease. The same process could occur with sex hormones, because they also influence growth. Therefore, to assess the possibility of reverse causality or confounding by growth, we also carried out sensitivity analyses by additionally adjusting for the imputed height or BMI variable (in quartiles according to sex). Missing values for BMI (*n* = 366) and height (*n* = 348) were imputed according to available data for child sex, frequency of exercise per week of the children, and anthropometric measures of the children and mothers (see Table S1). No remarkable changes in results were found when restricting analyses to individuals with available data for these two variables (data not shown).

In all the analyses, we excluded highly influential observations detected by examination of graphical representation of dfbetas (range, 0–3 observations excluded). We used the statistical software package STATA for all statistical analyses, and specifically the “intreg” command for interval (censored) regression (STATA Statistical Software, Release 12), and the statistical software R.3.1.1 (R Core Team 2014) for multiple imputation (*mice* package (van Buuren and Groothuis-Oudshoorn 2011). Where associations are referred to as statistically significant, this implies a *p <* 0.05.

## Results

Table 1 shows the characteristics of the study participants (*n* = 2,292) and Table 2 the summary figures for sex steroid hormones, IGF-1, and PFAS. A total of 96% of the children were white, the annual household family income was < $20,000 in 34% of them, and 51% were boys (Table 1). Levels of sex hormones and IGF-1 were slightly higher in girls than in boys. The proportion of observations in boys and girls above the LODs were 73% and 80% for estradiol, 69% and 75% for total testosterone, 19% and 28% for free testosterone, and 99.9% and 100% for IGF-1 (Table 2). Median concentrations of PFHxS, PFOA, PFOS, and PFNA in boys were 8, 35, 22, 1.7 ng/mL, and in girls they were 7, 30, 21, 1.7 ng/mL, respectively (Table 2). In boys and girls, correlations between the four PFAS were low (range of *r* = –0.08 and 0.33), except for PFOS versus PFHxS (*r* = 0.56 and 0.61 in boys and girls) (see Table S2).

**Table 1:** C8 Health Study Participants (*n* = 2,292), Mid-Ohio Valley (USA), 2005–2006.

Variable	Boys (*n* = 1,169)	Girls (*n* = 1,123)
Race/ethnicity (white)	1,120 (95.8)	1,075 (95.7)
Household income (< $20,000)	334 (33.9)	336 (34.9)
Age (years)	8.3 (7.3, 9.3)	8.4 (7.3, 9.3)
Body mass index (kg/m^2^)	17.9 (15.6, 20.8)	17.4 (15.3, 20.5)
Height (inches)	51.0 (48.0, 54.0)	50.0 (48.0, 53.5)
Values are *n* (%) or median (interquartile range). The *n* (%) of missing values in boys and girls was as follows: household annual family income: 184 (16%) and 160 (14%); body mass index: 179 (15%), and 187 (17%); and height: 168 (14%) and 180 (16%). Missing values were not considered for descriptive analyses.		

**Table 2:** Levels of sex hormones, IGF-1 and PFAS in children 6–9 years years of age (*n* = 2,292), Mid-Ohio Valley (USA), 2005–2006.

Hormone, IGF-1, and PFAS	LOD	Boys (*n* = 1,169)		Girls (*n* = 1,123)	
		*n* > LOD (%)	Median (IQR)	*n* > LOD (%)	Median (IQR)
Estradiol (pg/mL)	7	846 (72.6)	10 (< LOD, 15)	896 (80.0)	12 (LOD, 17)
Total testosterone (ng/dL)	10	809 (69.2)	13 (< LOD, 18)	840 (74.8)	15 (< LOD, 21)
Free testosterone (pg/mL)	0.2	216 (19.0)	< LOD (< LOD, 15)	313 (28.0)	< LOD (< LOD, 17)
IGF-1 (ng/mL)	25	1,159 (99.9)	147 (116, 187)	1,112 (100)	185 (142, 234)
PFHxS (ng/mL)	0.5	1,166 (99.7)	8.1 (4.2, 17.1)	1,120 (99.7)	7.0 (3.8, 13.8)
PFOA (ng/mL)	0.5	1,169 (100)	34.8 (15.3, 82.2)	1,123 (100)	30.1 (13.5, 74.0)
PFOS (ng/mL)	0.5	1,166 (99.7)	22.4 (16.5, 32.0)	1,123 (100)	20.9 (15.3, 29.4)
PFNA (ng/mL)	0.5	1,162 (99.4)	1.7 (1.3, 2.3)	1,119 (99.6)	1.7 (1.3, 2.4)
Abbreviations: IGF-1, insulin-like growth factor-1; IQR, interquartile range; LOD, detection limit; PFAS, perfluoroalkyl substances; PFHxS, perfluorohexane sulfonate; PFNA, perfluorononanoic acid; PFOA, perfluorooctanoate; PFOS, perfluorooctane sulfonate. The *n* (%) of missing values in boys and girls was as follows: estradiol: 3 (0.26%) and 3 (0.27%); total testosterone: 0 (0%) and 0 (0%); free testosterone: 5 (0.43%) and 5 (0.45%); and IGF-1: 9 (0.77%) and 11 (0.98%). Missing values were not considered for descriptive analyses.					

**Figure 1:**
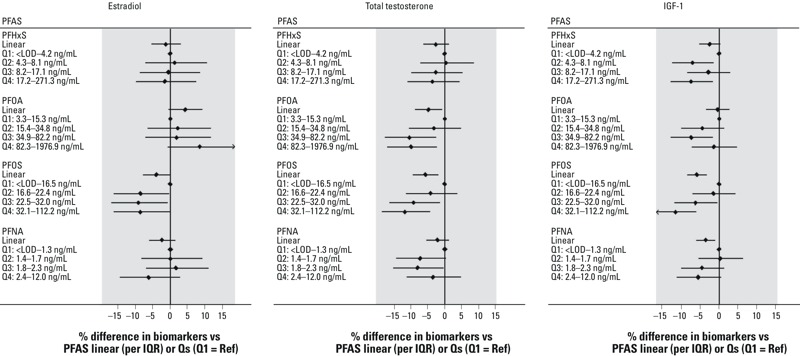
Difference in levels of sex hormones and IGF-1 in relation to PFAS (continuous or quartiles) in boys 6–9 years of age (*n *= 1,169), Mid-Ohio Valley (USA), 2005–2006. Abbreviations: IGF-1, insulin-like growth factor-1; PFAS, perfluoroalkyl substances; PFHxS, perfluorohexane sulfonate; PFNA, perfluorononanoic acid; PFOA, perfluorooctanoate; PFOS, perfluorooctane sulfonate; Q, quartile; Ref, reference. Models were adjusted for age and month of sampling. In addition, testosterone models were adjusted for time of sample ­collection. Estimates and 95% confidence intervals are presented. *p*-Trend was < 0.05 for PFOA or PFOS and total testosterone, and PFOS or PFNA and IGF-1.

**Figure 2:**
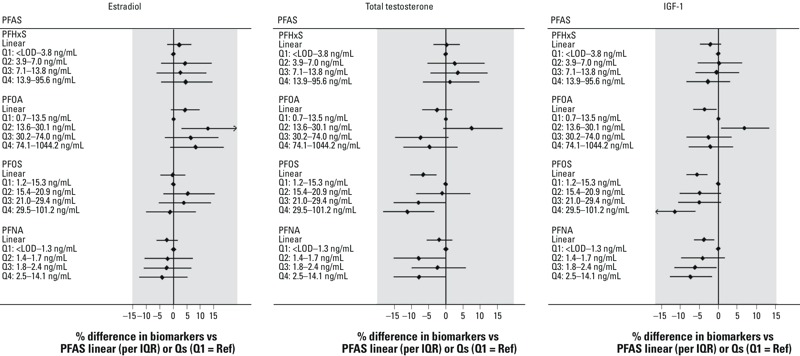
Difference in levels of sex hormones and IGF-1 in relation to PFAS (continuous or quartiles) in girls 6–9 years of age (*n *= 1,123), Mid-Ohio Valley (USA), 2005–2006. Abbreviations: IGF-1, insulin-like growth factor-1; PFAS, perfluoroalkyl substances; PFHxS, perfluorohexane sulfonate; PFNA, perfluorononanoic acid; PFOA, perfluorooctanoate; PFOS, perfluorooctane sulfonate; Q, quartile; Ref, reference. Models were adjusted for age and month of sampling. In addition, testosterone models were adjusted for time of sample ­collection. Estimates and 95% confidence intervals are presented. *p*-Trend was < 0.05 for PFOS and total testosterone or IGF-1, and PFNA and IGF-1.

Associations between PFAS and outcomes in children are shown in Table 3 and Figures 1–2 (see also Tables S3–S6). Any difference in effect by sex was assessed through interaction terms between sex and the contaminants in joint models (including boys and girls). The interaction with sex was not statistically significant except for PFOS and estradiol (*p* = 0.048) (Table 3). In the stratified models for boys, significant inverse associations were observed for PFOA and total testosterone (–4.9%; 95% CI: –8.7, –0.8%), PFOS and estradiol (–4.0%; 95% CI: –7.7, –0.1%), PFOS and total testosterone (–5.8%; 95% CI: –9.4, –2.0%), PFOS and IGF-1 (–5.9%; 95% CI: –8.3, –3.3%), and PFNA and IGF-1 (–3.5%; 95% CI: –6.0, –1.0%) (Table 3 and Figure 1). The magnitudes of the estimates were similar after adjustment for other PFAS or after including height or BMI in the models (see Table S3). Associations between each PFAS (in quartiles) and outcomes in boys are shown in Figure 1 (see also Table S5) where the lowest quartile is the reference exposure category. The decrease across PFAS quartiles was most clearly monotonic in the case of PFOS and both total testosterone (*p*-trend across quartiles = 0.002) and IGF-1 (*p*-trend < 0.001) and less clearly for PFNA and IGF-1 (*p*-trend = 0.031) (see Table S5).

**Table 3:** Difference in levels of sex hormones and IGF-1 in relation to PFAS concentrations in children 6–9 years of age (*n* = 2,292), Mid-Ohio Valley (USA), 2005–2006.

PFAS	Stratified, boys (*n* = 1,169) [% difference (95% CI)]^*a*^	Stratified, girls (*n* = 1,123) [% difference (95% CI)]^*a*^	Joint *p*-inter^*b*^
Association with ln(estradiol)^*c*^
PFHxS	–1.3 (–5.5, 3.1)	2.1 (–2.2, 6.5)	0.165
PFOA	4.3 (–0.4, 9.1)	4.2 (–0.7, 9.4)	0.924
PFOS	–4.0 (–7.7, –0.1)	–0.3 (–4.6, 4.2)	0.048
PFNA	–2.5 (–6.2, 1.4)	–2.4 (–6.3, 1.7)	0.622
Association with ln(total testosterone)^*d*^
PFHxS	–2.7 (–6.4, 1.2)	0.2 (–3.5, 4.0)	0.252
PFOA	–4.9 (–8.7, –0.8)	–2.5 (–6.7, 1.8)	0.425
PFOS	–5.8 (–9.4, –2.0)	–6.6 (–10.1, –2.8)	0.531
PFNA	–2.1 (–5.5, 1.3)	–1.9 (–5.5, 1.9)	0.700
Association with ln(IGF-1)^*c*^
PFHxS	–2.5 (–5.2, 0.3)	–2.1 (–4.8, 0.7)	0.829
PFOA	–0.4 (–3.4, 2.7)	–3.6 (–6.6, –0.5)	0.057
PFOS	–5.9 (–8.3, –3.3)	–5.6 (–8.2, –2.9)	0.761
PFNA	–3.5 (–6.0, –1.0)	–3.8 (–6.4, –1.2)	0.829
Abbreviations: CI, confidence interval; IGF-1, insulin-like growth factor-1; PFAS, perfluoroalkyl substances; PFHxS, perfluorohexane sulfonate; PFNA, perfluorononanoic acid; PFOA, perfluorooctanoate; PFOS, perfluorooctane sulfonate. ^***a***^Percent difference in outcome in relation to 75th vs. 25th percentile of ln(PFAS), derived from multiple linear regression model of outcome on ln(PFAS). The interquartile ranges in boys were 1.40, 1.68, 0.66, and 0.57 for ln(PFHxS), ln(PFOA), ln(PFOS), and ln(PFNA), respectively. Corresponding data in girls were 1.29, 1.70, 0.65, and 0.61. ^***b***^*p*-Value from the interaction term between PFAS and sex in joint models.^***c***^Models adjusted for age and month of sampling. ^***d***^Model adjusted for age, month, and time of sampling.			

In the stratified models for girls (Table 3 and Figure 2), significant inverse associations were found for PFOS and total testosterone (–6.6%; 95% CI: –10.1, –2.8%), PFOS and IGF-1 (–5.6%; 95% CI: –8.2, –2.9%), and PFNA and IGF-1 (–3.8%; 95% CI: –6.4, –1.2%). For PFOA, an association with IGF-1 was also found (–3.6%; 95% CI: –6.6, –0.5%) (Table 3), but this appeared to be an artifact of the raised mean IGF-1 in the second quartile (Figure 2; see also Table S6). Associations were similar after adjustment for other PFAS and results were also generally consistent when height or BMI was included (see Table S4). Across PFAS quartiles (Figure 2; see also Table S6), we found a more clearly monotonic decrease in total testosterone or IGF-1 in relation to concentrations of PFOS (*p*-trend across quartiles = 0.002 and < 0.001, respectively), and IGF-1 and PFNA (*p*-trend = 0.021).

## Discussion

We found serum PFAS concentrations to be associated with lower levels of sex hormones and IGF-1 in children 6–9 years of age. Across sexes, PFOS exposure was found to be more clearly related to the outcomes in young children. Concerning outcomes, IGF-1 was the most consistently associated with PFAS exposures.

In boys 6–9 years of age, serum PFOS concentrations were associated with lower estradiol, total testosterone, and IGF-1 levels. PFOA was also inversely associated with total testosterone, and PFNA with IGF-1. A statistically significant delay in the age at which boys started pubertal development (defined by testosterone levels > 50 ng/dL) was found for PFOS but not PFOA in the 8- to 18-year group of the same population (Lopez-Espinosa et al. 2011), supporting the current findings. Although the age of hormone measurement is not the same, and comparisons ought to be made with caution, our results in young children are also consistent with inverse associations between PFOS (median, 7.79 ng/mL) and testosterone in Danish men with a mean age of 19.6 years (Joensen et al. 2013). In contrast, no significant associations between PFOS and these hormones were found in a study of Taiwanese adolescent boys 12–17 years of age (Tsai et al. 2015) or in adult men from different European countries (median range, 7.6–44.7 ng/mL) (Specht et al. 2012; Vested et al. 2013). Concerning PFOA, neither an epidemiological study in adolescent boys (Tsai et al. 2015) nor those conducted in adult men with background or with high occupational PFOA exposure (median range, 3.02 ng/mL–5.71 μg/mL) found statistically significant associations with estradiol or testosterone (Costa et al. 2009; Joensen et al. 2013; Raymer et al. 2012). PFHxS and PFNA have been more rarely studied (Joensen et al. 2013; Tsai et al. 2015), and only one study found an inverse association between PFNA (median, 1.07 ng/mL) and estradiol in young men (Joensen et al. 2013). PFOS and PFNA levels in young boys were also associated with IGF-1 in the present study, but, to our knowledge, there are no previous epidemiological studies with which to compare our results, and this observation should be investigated in other populations.

In girls 6–9 years of age, we found PFOS to be associated with lower total testosterone and IGF-1 levels. PFNA was also inversely associated with IGF-1. There was no significant association between any of the four PFAS that were studied and estradiol levels. In previous studies, testosterone was found to be inversely associated with PFOS among Taiwanese girls 12–17 years of age (Tsai et al. 2015). In contrast, prenatal concentrations of PFHxS, PFOA and PFOS (medians, 1.6, 3.6, and 19.2 ng/mL, respectively) were positively associated with testosterone in girls 15 years of age from the ALSPAC cohort (UK) (Maisonet et al. 2015). Neither estradiol nor testosterone measured in 20-year-old Danish women (Kristensen et al. 2013) was statistically significantly associated with *in utero* PFOA or PFOS concentrations (medians, 3.6 and 21.1 ng/mL, respectively). To our knowledge, the association between IGF-1 and PFOS or PFNA observed in the present study has not been reported previously in girls either and needs to be further investigated.

Levels of sex hormones and IGF-1 during the prepubertal period or around onset of puberty are associated with age at menarche (Apter and Vihko 1985; Thankamony et al. 2012). Therefore, our findings of a relatively modest decrease in levels of testosterone and IGF-1 in girls who had not reported their first period yet might be of concern, because later menarche attainment was associated with exposure to these contaminants in two previous investigations. A 5.3-month delay in the age of menarche between the lowest and highest tertile of prenatal PFOA exposure was reported in young Danish women (Kristensen et al. 2013). We also found a 4.3- and 4.6-month delay in the age of menarche when comparing the 1st and 4th quartiles of PFOA and PFOS concentrations in the 8- to 18-year-old girls of the C8 Health Project (Lopez-Espinosa et al. 2011). Our study was cross-sectional, so physiological changes, including menstrual blood loss, could have affected PFAS excretion and thus serum concentrations. In fact, a pharmacokinetic model (Wu et al. 2015) has recently indicated that growth dilution and excretion of the contaminants through menstruation may cause lower PFAS concentrations in serum. This might explain, in part, the association between higher PFAS concentrations and older age of menarche in our previous study (Lopez-Espinosa et al. 2011). Finally, a nested case–control study conducted in the ALSPAC cohort did not find a significant association between cord blood PFAS concentrations and the age of menarche (Christensen et al. 2011).

The possible biological mechanisms underlying the potential effects of PFAS on decreasing levels of sex hormones and IGF-1 are still not well understood, but several modes of action may be involved. Thus, some animal evidence exists on their possible effects on androgen synthesis. Concerning PFOA-exposed male rodents, a decrease in testosterone production was found to be due to the reduction in the conversion of 17α-hydroxyprogesterone to androstenedione (Cook et al. 1992) and to the inhibition of 3β- and 17β-hydroxysteroid dehydrogenase activities in Leydig cells (Zhao et al. 2010). For PFOS, the decrease in testosterone levels was attributed to a reduction in the expression levels of testicular receptors for FSH, LH, GH, IGF-1, and inhibin subunits as well as to the impairment of testicular steroidogenesis in PFOS-exposed male mice (Wan et al. 2011) and to the impairment of fetal Leydig cells in male fetus of prenatally PFOS exposed rats (Zhao et al. 2014). PFAS could also interfere with the androgen or estrogen pathway by disturbing the expression of genes associated with the metabolism of steroid hormones, as reported in liver tissue from PFOA-exposed mouse fetuses (Rosen et al. 2007) or decreasing the expression of genes related to the hypothalamic–pituitary–thyroid (HPT) axis, the growth hormone–insulin-like growth factor axis (GH–IGF), and the steroid hormone axis, as reported in salmon embryos and larvae exposed to PFOA and PFOS (Spachmo and Arukwe 2012).

The main strengths of the present study are the large sample size (over 2,000 participants) and the several individual potential confounders considered in our models. It is believed that these participants are representative, given the high participation rates in the C8 Health Project (77% in the 5–10 year age group) (Frisbee et al. 2009) among those children who drank contaminated water in the Mid-Ohio Valley, and this diminishes concern about potential selection biases. Notably, the strongest significant associations were found for PFOS, whose concentrations in the Mid-Ohio Valley participants were similar to the general U.S. population, assessed in the 2005–2006 National Health and Nutrition Examination Survey (NHANES) (Kato et al. 2011). Although NHANES information in children 6–9 years of age was not available, median concentrations of PFOS were 15 ng/mL in NHANES children 12–19 years of age versus 20 ng/mL in 2005–2006 C8 Health Project children 11–17 years of age (Lopez-Espinosa et al. 2012). However, patterns of human exposure to PFOS are changing worldwide as a consequence of changes in regulatory policies and PFOS exposure levels have decreased in the United States (Kato et al. 2011). Therefore, the levels reported in the present study may not be representative of current serum levels.

The 6–9 years age group was selected to investigate possible alterations in biomarkers involved in sexual maturation and growth due to PFAS exposure before or around the onset of puberty (i.e. entry to Tanner stage 2). We excluded boys who had started puberty (considered by the authors as testosterone levels > 50 ng/dL) and girls with menarche, but we were unable to differentiate children in different pubertal Tanner stages. We presume that some of our children might be in Tanner stage 2 or higher since according to a study (*n* = 2,070; collection period, 2005–2010), 26.1% (95% CI: 19.5, 33.6) and 8.1% (95% CI: 4.4, 13.4) of American non-Hispanic white boys had experienced the onset of genital and pubic hair development, respectively, at the age of 9 years (Herman-Giddens et al. 2012). In American non-Hispanic white girls (*n* = 420; collection period, 2004–2011), the median age of onset of breast development was 9.7 years (Biro et al. 2013). Our cross-sectional design has different limitations. First, we cannot rule out the possibility that growth (and associated IGF-1 increases) may actually cause a reduction in these PFAS by diluting them in a growing body or by affecting excretion rates. We consider this unlikely, because adding height or BMI (as markers of growth) to the models made no difference to the associations. Second, our findings suggest a possible alteration of sex hormones and IGF-1 due to PFAS exposure at 6–9 years of age, which is a period of high vulnerability to the possible effects of exposure to chemicals with endocrine properties, such as the PFAS. But apart from this period of life, there are also others, such as the fetal stage, in which the development of the hypthalamic–pituitary–gonadal (HPG), HPT, or GH-IGF axes, ovaries, and testicles could have been negatively affected at any level. Third, we had a single measure of the levels of these biomarkers, which are constantly changing in this period. In fact, their levels gradually increase from mid-childhood, rise sharply in puberty and decline steadily with age for IGF-1 (Pollak 2000), and continue to rise after puberty for sex hormones (Cole et al. 2015; Garnett et al. 2004). Therefore, further longitudinal studies need to be conducted to examine several periods of vulnerability. Another weakness of the present study is the lack of other markers of growth or pubertal development such as LH, FSH, or GH, which would have yielded more comprehensive information concerning the functioning of the HPG and GH-IGF axes. Finally, serum testosterone levels exhibit variable diurnal cyclicity (Ankarberg and Norjavaara 1999; Ankarberg-Lindgren and Norjavaara 2004), but the adjustment of models by the timing of sample collection enabled us to limit potential confounding from diurnal fluctuations.

## Conclusions

Our findings show PFOS and PFNA concentrations to be associated with lower levels of IGF-1 in boys and girls 6–9 years of age. Our results also suggest an inverse association between PFAS and sex hormones with a decrease in testosterone in both boys and girls for PFOS and for PFOA in boys. PFOS was also inversely associated with estradiol only in boys. PFOS appears to be the most active of the four compounds studied in affecting sex hormones and IGF-1 in young children.

## Supplemental Material

(258 KB) PDF
