# Supplementary material for: Perfluoroalkyl Substances, Sex Hormones, and Insulin-like Growth Factor-1 at 6–9 Years of Age: A Cross-Sectional Analysis within the C8 Health Project
Source: Environ Health Perspect. 2016 Jan 22;124(8):1269–75. doi: 10.1289/ehp.1509869 (PMC4977043; doi:10.1289/ehp.1509869)
Supplement: (258 KB) PDF [file ehp.1509869.s001.acco.pdf]

Environ Health Perspect
DOI: 10.1289/ehp.1509869

Note to readers with disabilities: *EHP* strives to ensure that all journal content is accessible to all readers. However, some figures and Supplemental Material published in *EHP* articles may not conform to 508 standards due to the complexity of the information being presented. If you need assistance accessing journal content, please contact ehp508@niehs.nih.gov. Our staff will work with you to assess and meet your accessibility needs within 3 working days.

Supplemental Material

Perfluoroalkyl Substances, Sex Hormones, and Insulin-like Growth Factor-1 at 6–9 Years of Age: A Cross-Sectional Analysis within the C8 Health Project

Maria-Jose Lopez-Espinosa, Debapriya Mondal, Ben G. Armstrong, Brenda Eskenazi, and Tony Fletcher

Table of Contents

Table S1. Number (%) of imputed values and regression model for each variable (*n*: 2,292 cases), Mid-Ohio Valley (USA), 2005-2006
Table S2. Pearson's correlations between ln(PFAS) in children aged 6-9 years, Mid-Ohio Valley (USA), 2005-2006
Table S3. Difference in levels of sex hormones and IGF-1 in relation to PFAS in boys aged 6-9 years, Mid-Ohio Valley (USA), 2005-2006
Table S4. Difference in levels of sex hormones and IGF-1 in relation to PFAS in girls aged 6-9 years, Mid-Ohio Valley (USA), 2005-2006
Table S5. Difference in levels of sex hormones and IGF-1 by PFAS quartiles in boys aged 6-9 years, Mid-Ohio Valley (USA), 2005-2006
Table S6. Difference in levels of sex hormones and IGF-1 by PFAS quartiles in girls aged 6-9 years, Mid-Ohio Valley (USA), 2005-2006
References

Table S1. Number (%) of imputed values and regression model for each variable (*n*: 2,292 cases), Mid-Ohio Valley (USA), 2005-2006

**Outcome variables**[a]	**Imputed**		**Method**
Ln(estradiol)	6	(0.3)	Linear regression
Ln(total testosterone)	0	(0.0)	-
Ln(IGF-1)	20	(0.9)	Linear regression
**Exposure variables**			
Ln(PFHxS)	0	(0.0)	-
Ln(PFOA)	0	(0.0)	-
Ln(PFOS)	0	(0.0)	-
Ln(PFNA)	0	(0.0)	-
**Covariates**			
Age	0	(0.0)	-
Sampling month	0	(0.0)	-
Sampling hour	1	(0.0)	Predictive mean matching
BMI[b]	366	(16.0)	Linear regression
Height[b]	348	(15.2)	Linear regression
Weight[b]	50	(2.2)	Linear regression
Household income	344	(15.0)	Ordered logistic regression
Race	0	(0.0)	-
Sex	0	(0.0)	-
**Other variables (not used in main analysis)**			
Maternal education	1,208	(52.7)	Ordered logistic regression
Maternal current employment	1,207	(52.7)	Ordered logistic regression
Maternal race	1,207	(52.7)	Logistic regression
Child's frequency of exercise	1,485	(64.8)	Ordered logistic regression

[a]In order to define a single imputed dataset (*n* = 2,292), missing values in the outcome variables were multiple-imputed. Subsequently, these values were deleted before analysis and recombination. [b]Body mass index (BMI), height, and weight, respectively, transformed into a z-score based on the 2000 U.S. Center for Disease Control and Prevention growth charts of BMI/height/weight-for-age (CDC EpiInfo 2010).

Table S2. Pearson's correlations between ln(PFAS) in children aged 6-9 years, Mid-Ohio Valley (USA), 2005-2006

**PFAS**	**Boys (*n* = 1,169)**				**Girls (*n* = 1,123)**			
	**PFHxS**	**PFOA**	**PFOS**	**PFNA**	**PFHxS**	**PFOA**	**PFOS**	**PFNA**
**PFOA**	0.17	1			0.16	1		
**PFOS**	0.56	0.23	1		0.61	0.23	1	
**PFNA**	0.13	-0.09	0.25	1	0.18	-0.08	0.33	1

$p \leq 0.001$ in all Pearson’s correlations

Table S3. Difference in levels of sex hormones and IGF-1 in relation to PFAS in boys aged 6-9 years, Mid-Ohio Valley (USA), 2005-2006

PFAS	Main analyses[a]	Adding other PFAS[b]	Adding height[c]	Adding BMI[d]
	%difference (95% CI)[e]	%difference (95% CI)[e]	%difference (95% CI)[e]	%difference (95% CI)[e]
**Association with ln(estradiol)**				
PFHxS	-1.3 (-5.5, 3.1)	1.8 (-3.4, 7.3)	-1.1 (-5.3, 3.2)	-0.9 (-5.1, 3.5)
PFOA	4.3 (-0.4 , 9.1)	5.3 (0.4, 10.3)	4.6 (0.0, 9.5)	4.5 (-0.1, 9.3)
PFOS	-4.0 (-7.7, -0.1)	-5.6 (-10.2, -0.7)	-3.7 (-7.4, 0.2)	-3.3 (-7.1, 0.6)
PFNA	-2.5 (-6.2, 1.4)	-0.9 (-4.8, 3.2)	-2.2 (-5.9, 1.7)	-2.4 (-6.1, 1.4)
**Association with ln(total testosterone)**				
PFHxS	-2.7 (-6.4, 1.2)	1.1 (-3.7, 6.1)	-2.4 (-6.1, 1.5)	-2.0 (-5.7, 1.9)
PFOA	-4.9 (-8.7, -0.8)	-4.0 (-8.0, 0.1)	-4.8 (-8.6, -0.8)	-4.3 (-8.1, -0.3)
PFOS	-5.8 (-9.4, -2.0)	-5.3 (-10.0, -0.3)	-5.0 (-8.6, -1.2)	-4.7 (-8.4, -1.0)
PFNA	-2.1 (-5.5, 1.3)	-0.9 (-4.5, 2.8)	-1.7 (-5.1, 1.8)	-1.9 (-5.2, 1.5)
**Association with ln(IGF-1)**				
PFHxS	-2.5 (-5.2, 0.3)	1.5 (-2.0, 5.0)	-2.0 (-4.7, 0.8)	-1.8 (-4.5, 1.0)
PFOA	-0.4 (-3.4, 2.7)	0.9 (-2.2, 4.0)	-0.4 (-3.3, 2.6)	0.0 (-3.0, 3.0)
PFOS	-5.9 (-8.3, -3.3)	-5.9 (-8.9, -2.7)	-5.0 (-7.5, -2.5)	-5.0 (-7.5, -2.4)
PFNA	-3.5 (-6.0, -1.0)	-2.3 (-4.9, 0.3)	-2.9 (-5.3, -0.4)	-3.4 (-5.8, -0.9)

BMI, body mass index; CI, confidence interval; IGF-1, insulin growth factor-1; PFAS, perfluoroalkyl substances; PFHxS, perfluorohexane sulfonate; PFNA, perfluorononanoic acid; PFOA, perfluorooctanoate; PFOS, perfluorooctane sulfonate.

[a]Models adjusted for age, and month of sampling (estradiol, testosterone, and IGF-1), and time of sampling (testosterone). In addition, models adjusted for the other three ln(PFAS)[b] or BMI[c] or height[d]. [e]%difference in outcome in relation to 75th vs. 25th percentile of ln(PFAS), derived from multiple linear regression model of outcome on ln(PFAS). The interquartile ranges in boys were 1.40, 1.68, 0.66, and 0.57 for ln(PFHxS), ln(PFOA), ln(PFOS), and ln(PFNA), respectively.

Table S4. Difference in levels of sex hormones and IGF-1 in relation to PFAS in girls aged 6-9 years, Mid-Ohio Valley (USA), 2005-2006

PFAS	Main analyses[a]	Adding other PFAS[b]	Adding height[c]	Adding BMI[d]
	%difference (95% CI)[e]	%difference (95% CI)[e]	%difference (95% CI)[e]	%difference (95% CI)[e]
**Association with ln(estradiol)**				
PFHxS	2.1 (-2.2,6.5)	2.6 (-2.6, 8.1)	2.4 (-1.9, 6.8)	2.2 (-2.0, 6.7)
PFOA	4.2 (-0.7, 9.4)	3.8 (-1.1, 9.0)	4.2 (-0.7, 9.4)	4.4 (-0.5, 9.6)
PFOS	-0.3 (-4.6, 4.1)	-1.1 (-6.7, 4.7)	-0.1 (-4.4, 4.4)	0.6 (-3.8, 5.2)
PFNA	-2.4 (-6.3, 1.7)	-2.3 (-6.5, 2.1)	-2.0 (-5.9, 2.1)	-2.0 (-5.9, 2.0)
**Association with ln(total testosterone)**				
PFHxS	0.2 (-3.5, 4.0)	7.2 (2.3, 12.3)	0.5 (-3.2, 4.4)	0.3 (-3.3, 4.1)
PFOA	-2.5 (-6.7, 1.8)	-1.1 (-5.4, 3.4)	-2.2 (-6.4, 2.2)	-1.7 (-5.8, 2.7)
PFOS	-6.6 (-10.1, -2.8)	-11.1 (-15.6, -6.3)	-6.2 (-9.8, -2.5)	-5.4 (-9.0, -1.6)
PFNA	-1.9 (-5.5, 1.9)	1.1 (-2.9, 5.3)	-1.5 (-5.1, 2.3)	-1.4 (-5.0, 2.3)
**Association with ln(IGF-1)**				
PFHxS	-2.1 (-4.8, 0.7)	1.9 (-1.5, 5.4)	-1.6 (-4.3, 1.1)	-1.8 (-4.5, 0.9)
PFOA	-3.6 (-6.6, -0.5)	-2.6 (-5.7, 0.5)	-3.4 (-6.4, -0.4)	-3.4 (-6.3, -0.3)
PFOS	-5.6 (-8.2, -2.9)	-5.7 (-9.1, -2.1)	-5.2 (-7.8, -2.6)	-4.8 (-7.4, -2.1)
PFNA	-3.8 (-6.4, -1.2)	-2.2 (-5.0, 0.7)	-3.2 (-5.7, -0.5)	-3.4 (-6.0, -0.8)

BMI, body mass index; CI, confidence interval; IGF-1, insulin growth factor-1; PFAS, perfluoroalkyl substances; PFHxS, perfluorohexane sulfonate; PFNA, perfluorononanoic acid; PFOA, perfluorooctanoate; PFOS, perfluorooctane sulfonate.

[a]Models adjusted for age, and month of sampling (estradiol, testosterone, and IGF-1), and time of sampling (testosterone). In addition, models adjusted for the other three ln(PFAS)[b] or BMI[c] or height[d]. [e]%difference in outcome in relation to 75th vs. 25th percentile of PFAS, derived from multiple linear regression model of outcome on ln(PFAS). The interquartile ranges in girls were 1.29, 1.70, 0.65, and 0.61 for ln(PFHxS), ln(PFOA), ln(PFOS), and PFNA, respectively.

Table S5. Difference in levels of sex hormones and IGF-1 by PFAS quartiles in boys aged 6-9 years, Mid-Ohio Valley (USA), 2005-2006

**PFAS**[a]	**Estradiol**[b]		**Total testosterone**[c]		**IGF-1**[b]	
	**%difference (95%CI)**[d]	***p*-trend**[e]	**%difference (95%CI)**[d]	***p*-trend**[e]	**%difference (95%CI)**[d]	***p*-trend**[e]
**PFHxS**		0.597		0.278		0.066
**Q1: <LOD-4.2**	Ref		Ref		Ref	
**Q2: 4.3-8.1**	1.2 (-7.2, 10.4)		0.3 (-7.2, 8.5)		-7.0 (-12.2, -1.5)	
**Q3: 8.2-17.1**	-0.5 (-8.8, 8.5)		-2.6 (-9.9, 5.3)		-2.9 (-8.3, 2.9)	
**Q4: 17.2-271.3**	-1.6 (-9.8, 7.4)		-3.7 (-11.0, 4.2)		-7.4 (-12.6, -1.8)	
**PFOA**		0.057		0.030		0.535
**Q1: 3.3-15.3**	Ref		Ref		Ref	
**Q2: 15.4-34.8**	2.1 (-6.5, 11.5)		-3.2 (-10.6, 4.7)		-4.4 (-9.8, 1.3)	
**Q3: 34.9-82.2**	1.8 (-7.1, 11.5)		-10.4 (-17.6, -2.6)		-7.4 (-12.8, -1.6)	
**Q4: 82.3-1976.9**	8.5 (-0.8, 18.5)		-10.0 (-17.0, -2.4)		-1.4 (-7.1, 4.6)	
**PFOS**		0.103		0.002		<0.001
**Q1: <LOD-16.5**	Ref		Ref		Ref	
**Q2: 16.6-22.4**	-8.6 (-16.2, -0.3)		-4.2 (-11.4, 3.6)		-1.5 (-7.1, 4.4)	
**Q3: 22.5-32.0**	-9.2 (-16.9, -0.8)		-9.2 (-16.1, -1.6)		-6.3 (-11.6, -0.6)	
**Q4: 32.1-112.2**	-8.6 (-16.3, -0.1)		-11.8 (-18.6, -4.3)		-11.5 (-16.6, -6.1)	
**PFNA**		0.120		0.822		0.031
**Q1:<LOD-1.3**	Ref		Ref		Ref	
**Q2: 1.4-1.7**	0.0 (-8.3, 9.1)		-7.3 (-14.3, 0.3)		0.3 (-5.4, 6.3)	
**Q3: 1.8-2.3**	1.7 (-6.9, 11.0)		-8.1 (-15.1, -0.4)		-4.6 (-10.0, 1.3)	
**Q4: 2.4-12.0**	-6.2 (-14.4, 2.7)		-3.5 (-11.1, 4.8)		-5.5 (-11.1, 0.4)	

CI, confidence interval; IGF-1, insulin growth factor-1; PFAS, perfluoroalkyl substances; PFHxS, perfluorohexane sulfonate; PFNA, perfluorononanoic acid; PFOA, perfluorooctanoate; PFOS, perfluorooctane sulfonate.

[a]PFAS in ng/mL.[b]Models adjusted for age and month of sampling. [c]Models adjusted for age, month, and time of sampling. [d]%difference in outcome across 4 quartiles of exposure (with the lowest as the reference). [e]*p* as a test of linear trend.

Table S6. Difference in levels of sex hormones and IGF-1 by PFAS quartiles in girls aged 6-9 years, Mid-Ohio Valley (USA), 2005-2006

**PFAS**[a]	**Estradiol**[b]		**Total testosterone**[c]		**IGF-1**[b]	
	**%difference (95%CI)**[d]	***p*-trend**[e]	**%difference (95%CI)**[d]	***p*-trend**[e]	**%difference (95%CI)**[d]	***p*-trend**[e]
**PFHxS**		0.495		0.991		0.266
**Q1: <LOD-3.8**	Ref		Ref		Ref	
**Q2: 3.9-7.0**	4.3 (-4.5, 13.8)		2.6 (-5.2, 11.1)		0.2 (-5.4, 6.1)	
**Q3: 7.1-13.8**	2.6 (-6.1, 12.0)		3.4 (-4.4, 12.0)		-0.5 (-6.1, 5.4)	
**Q4: 13.9-95.6**	4.4 (-4.4, 14.0)		1.2 (-6.5, 9.6)		-2.7 (-8.2, 3.0)	
**PFOA**		0.531		0.077		0.098
**Q1: 0.7-13.5**	Ref		Ref		Ref	
**Q2: 13.6-30.1**	12.6 (3.0, 23.1)		7.4 (-0.7, 16.3)		6.8 (0.8, 13.2)	
**Q3: 30.2-74.0**	6.2 (-3.0, 16.4)		-7.3 (-14.7, 0.8)		-2.6 (-8.3, 3.4)	
**Q4: 74.1-1044.2**	8.1 (-1.2, 18.4)		-4.7 (-12.2, 3.5)		-2.1 (-7.7, 3.9)	
**PFOS**		0.508		0.002		<0.001
**Q1: 1.2-15.3**	Ref		Ref		Ref	
**Q2: 15.4-20.9**	5.2 (-3.7, 14.9)		-1.1 (-8.6, 7.1)		-4.9 (-10.2, 0.7)	
**Q3: 21.0-29.4**	3.7 (-5.2, 13.4)		-7.8 (-15.0, -0.1)		-5.0 (-10.4, 0.7)	
**Q4: 29.5-101.2**	-1.3 (-9.9, 8.2)		-11.1 (-18.2, -3.5)		-11.4 (-16.5, -6.0)	
**PFNA**		0.392		0.158		0.021
**Q1: <LOD-1.3**	Ref		Ref		Ref	
**Q2: 1.4-1.7**	-2.2 (-10.6, 7.1)		-7.9 (-15.0, -0.1)		-4.1 (-9.6, 1.7)	
**Q3: 1.8-2.4**	-2.5 (-10.7, 6.6)		-2.4 (-9.8, 5.6)		-6.1 (-11.4, -0.5)	
**Q4: 2.5-14.1**	-4.2 (-12.5, 4.9)		-7.8 (-15.0, 0.1)		-7.3 (-12.6, -1.7)	

CI, confidence interval; IGF-1, insulin growth factor-1; PFAS, perfluoroalkyl substances; PFHxS, perfluorohexane sulfonate; PFNA, perfluorononanoic acid; PFOA, perfluorooctanoate; PFOS, perfluorooctane sulfonate.

[a]PFAS in ng/mL. [b]Models adjusted for age and month of sampling. [c]Models adjusted for age, month, and time of sampling. [d]%difference in outcome across 4 quartiles of exposure (with the lowest as the reference). [e]*p* as a test of linear trend.

References

CDC Epi Info. 2010. EpiInfo^{7M} 7. Version 3.5.2. Available: http://www.cdc.gov/epiinfo. [accessed 12 March 2011].
